# Supplementary material for: Structure and Chemical Composition of ca. 10-Million-Year-Old (Late Miocene of Western Amazon) and Present-Day Teeth of Related Species
Source: Biology (Basel). 2022 Nov 8;11(11):1636. doi: 10.3390/biology11111636 (PMC9687460; doi:10.3390/biology11111636)
Supplement: Supplementary file 1 [file biology-11-01636-s001.zip › biology-1882597-Supplementary Titles-23.11 edit.pdf]

“Structure and chemical composition of ca. 10-million-year-old (Late Miocene of western Amazon) and present-day teeth of related species”
Supplementary Material:

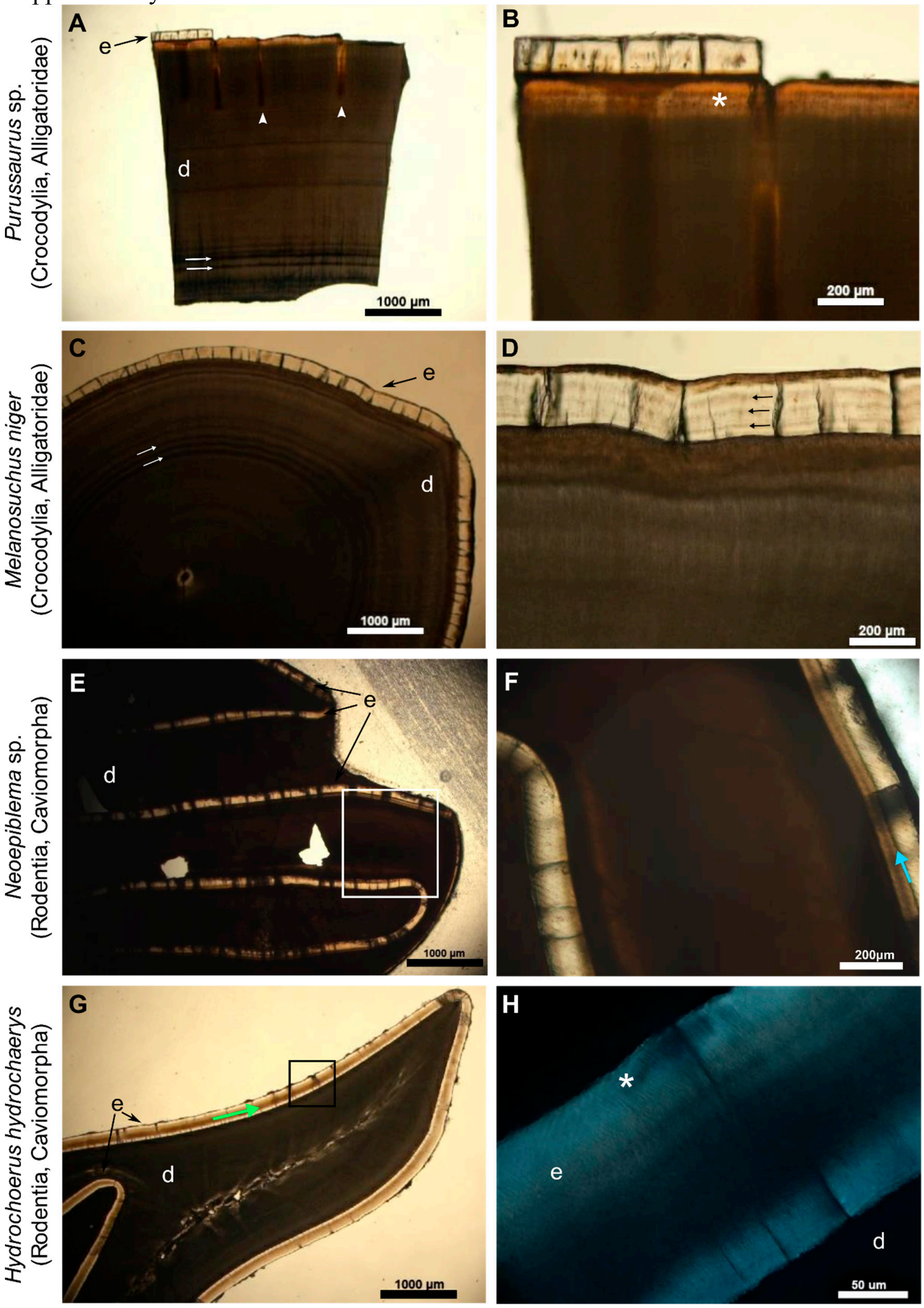

Figure S1 A photomicrograph panel of ground sections (~ 100 µm) of the 4 teeth used in this study. The images show that dental enamel (e) is translucent in all specimens, while dentine (d) is dark/opaque in all specimens. There is some superficial mineral loss in dentine of *Purussaurus sp.*, possibly due to acids from the environment (A: arrow head; B: asterisk). The white arrows of panels A

and C show marked apposition lines in the dentine. In D, black arrows in the enamel show appositional lines. In F the blue arrow indicates a very pronounced line. In G the *H. hydrochaeris* enamel is shown; the arrow indicates an area in middle enamel that seems different from the inner and the outer enamel. In H a larger magnification of the black square is shown under polarized light. Due to the thickness of this section, the prismatic aspect of this enamel is difficult to recognize, but in the outer enamel (asterisk) diagonal lines are enamel rods.

Table S1. ICP-MS method quantification limits (LOQ) of the elements in the study.

Element	LOQ (ng/g)	Element	LOQ (ng/g)	Element	LOQ (ng/g)	Element	LOQ (ng/g)	Element	LOQ (ng/g)
Zn (66)	0.1	Co (59)	0.05	Se (82)	0.08	Sm (152)	0.05	U (238)	0.008
Pb (208)	0.007	K (39)	0.1	Ba (138)	0.05	Ni (60)	0.01	Be (9)	0.001
Fe (54)	0.1	Cd (111)	0.009	La (139)	0.08	Pd (106)	0.001	Ag (107)	0.001
Mg (24)	0.1	Mn (55)	0.01	Cu (63)	0.1	U (235)	0.07	V (51)	0.05
Al (27)	0.06	Ce (140)	0.08	Th (232)	0.06	U (234)	0.22	Cr (53)	0.05
As(75)	0.09	Rb (85)	0.02	Bi (029)	0.03				

Table S2. **Twenty-seven (27) microelements in the enamel composition determined by ICP-MS.** The sample concentration results in ppb were converted to concentration (ppm) of enamel mass based on Calcium percentage values recovered by EDS-SEM. Purussaurus (1): Darkest region of enamel, (2) Predominant brown enamel, (3) Yellow region of enamel.

	Sample (µg/g) ppm					
**Element (Isotope)**	***Purussaurus* sp. (1)**	***Purussaurus* sp. (2)**	***Purussaurus* sp. (3)**	***M. niger***	***Neoepiblema* sp.**	***H. hydrochaeris***
**Zn (66)**	114836	-	44509	-	234297	96565
**Pb (208)**	36873	25110	10950	528	22306	860
**Fe (54)**	-	-	-	17053	14774	5872
**Mg (24)**	13468	-	7172	2602	14827	11696
**Al (27)**	9211	-	3990	642	14412	8233
**Co (59)**	8411	5630	2412	55	4926	114
**K (39)**	6761	5239	4471	4271	7416	1060
**Cd (111)**	3995	2731	1183	46	2334	82
**Mn (55)**	2650	1134	1715	1372	3285	93
**Ce (140)**	717	-	222	56	1514	38
**Se (82)**	386	-	20	-	72	177
**Ba (138)**	299	-	353	993	285	222
**La (139)**	228	-	64	23	576	26
**Cu (63)**	179	17	46	13	135	-
**Th (232)**	125	-	90	0.7	871	1.4
**Sm (152)**	109	-	41	4.6	177	-

**Ni (60)**	78	45	17	21	30	13
**Pd (106)**	36	1.0	8.3	-	139	-
**U (235)**	36	-	44	2.0	436	-
**U (238)**	10	-	4.8	0.01	176	-
**U (234)**	-	-	-	-	1224	-
**Be (9)**	13	3.0	5.8	3.2	26	-
**Tl (205)**	5.3	-	0.4	0.2	4.4	0.1
**Ag (107)**	2.3	20	3.2	0.03	26	-
**V (51)**	-	-	177	-	819	500
**Cr (53)**	-	-	721	-	2501	1798
**As (75)**	-	-	-	-	461	179
**Rb (85)**	-	-	1.2	12	-	-
**Bi (209)**	-	-	-	0.003	50	-

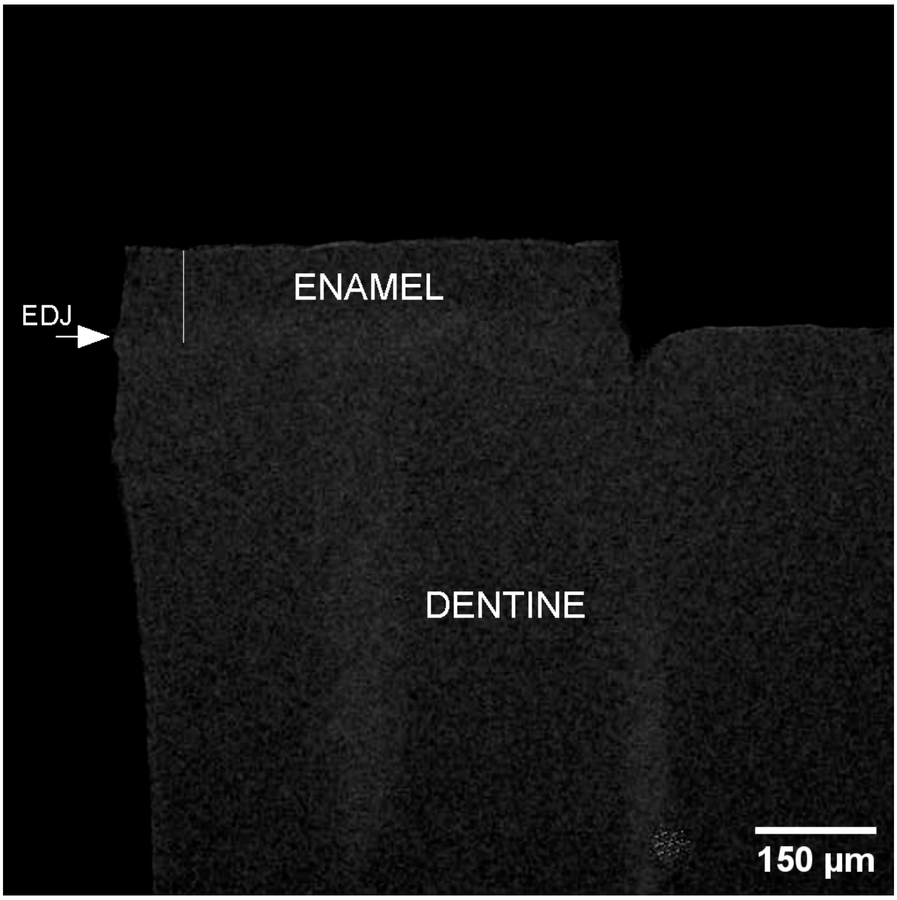

Figure S2. Microradiographic image of a ground section of dental enamel sample from *Purussaurus* sp. The arrow indicates the enamel-dentine junction (EDJ). Points of biochemical components (mineral, water, and organic) measurements (at 15, 35, 55, 75, and 95 µm from the enamel surface) were located along the longitudinal line shown in the enamel layer. The enamel thickness is 115 µm.

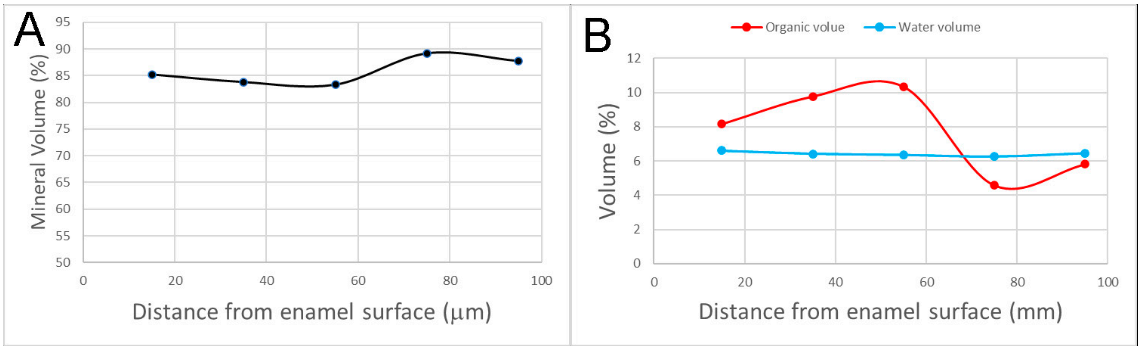

Figure S3. Mineral (A), organic (B), and total water (B) enamel component volumes of *Purussaurus* sp. at histological points located along the longitudinal line shown in Supplementary Figure 2.

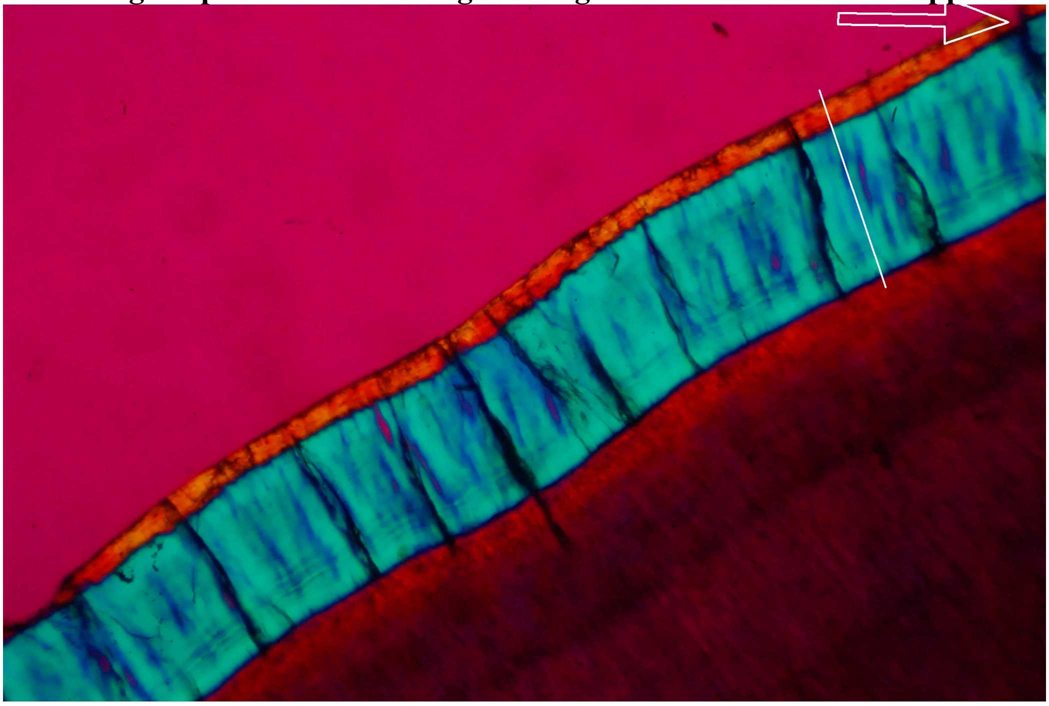

Figure S4. **Ground section of dental enamel sample from *Melanosuchus niger* under polarizing microscopy.** The image was taken with the Red I filter (most of the enamel is shown in blue). The ground section was submitted to water immersion before analysis. On the surface of the enamel there is a layer that is different from the underlying enamel (orange).

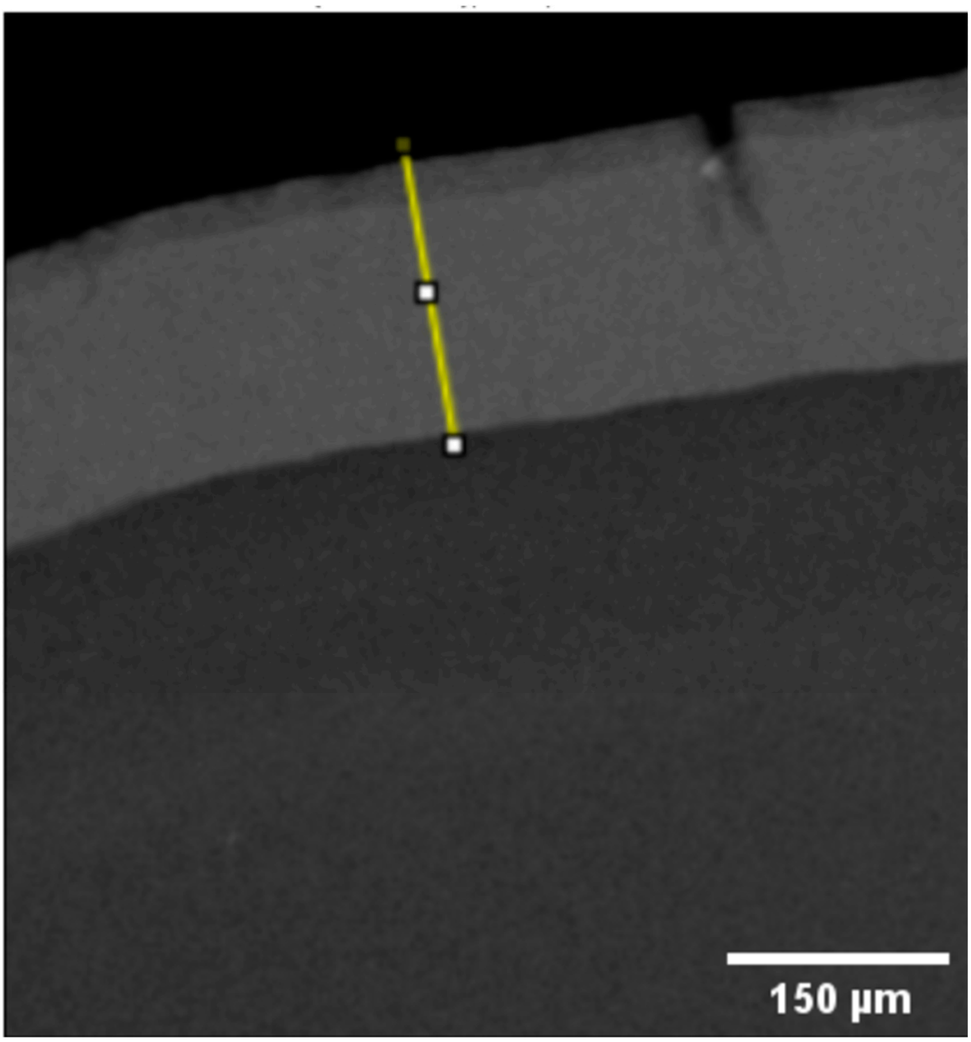

Figure S5. Microradiographic image of a ground section of dental enamel sample from *M. niger*. Points of biochemical components (mineral, water, and organic) measurements (at 15, 55, 95, 135, and 175 µm from the enamel surface) were located along the longitudinal line shown in the enamel layer. The enamel thickness is 190 µm.

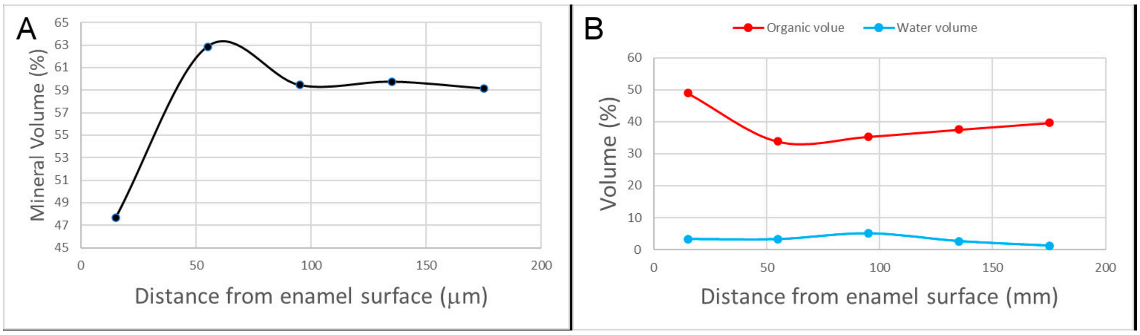

Figure S6. **Mineral (A), organic (B), and total water (B) enamel component volumes of *M. niger* at histological points located along the longitudinal line shown in Figure 5.**

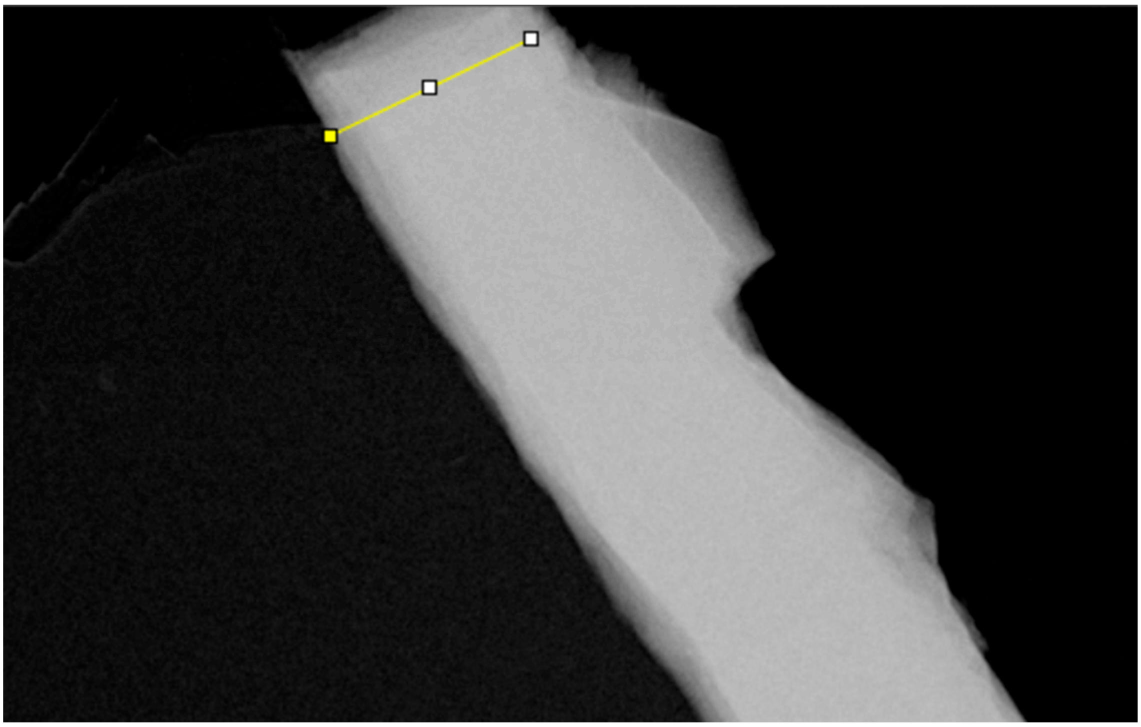

Figure S7. Microradiographic image of a ground section of dental enamel sample from *Neoepiblema* sp. Points of biochemical components (mineral, water, and organic)
measurements (at 40, 70, 100, 130, and 160 µm from the enamel surface) were located along the longitudinal line shown in the enamel layer. The enamel thickness is 205 µm.

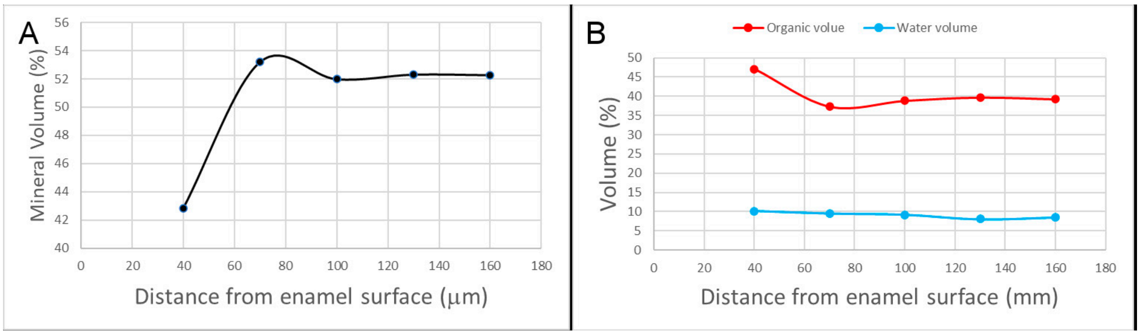

Figure S8. **Mineral (A), organic (B), and total water (B) enamel component volumes of *Neoepiblema* sp. at histological points located along the longitudinal line shown in Figure 7.**

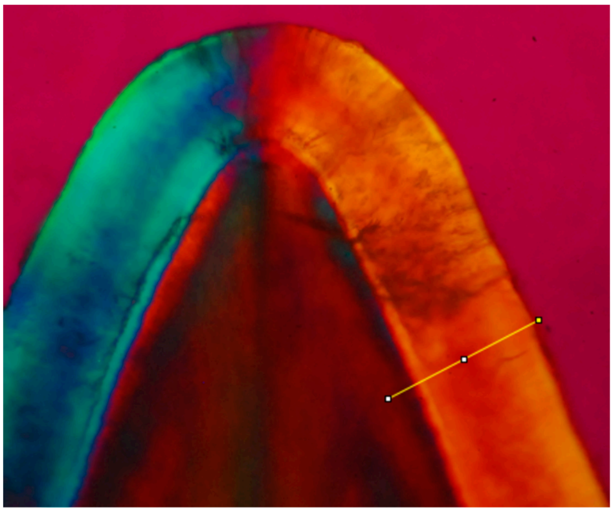

Figure S9. Image of a ground section of dental enamel sample from *Hydrochoerus hydrochaeris* under polarizing microscopy. The image was taken with the Red I filter. The ground section was submitted to water immersion before analysis.

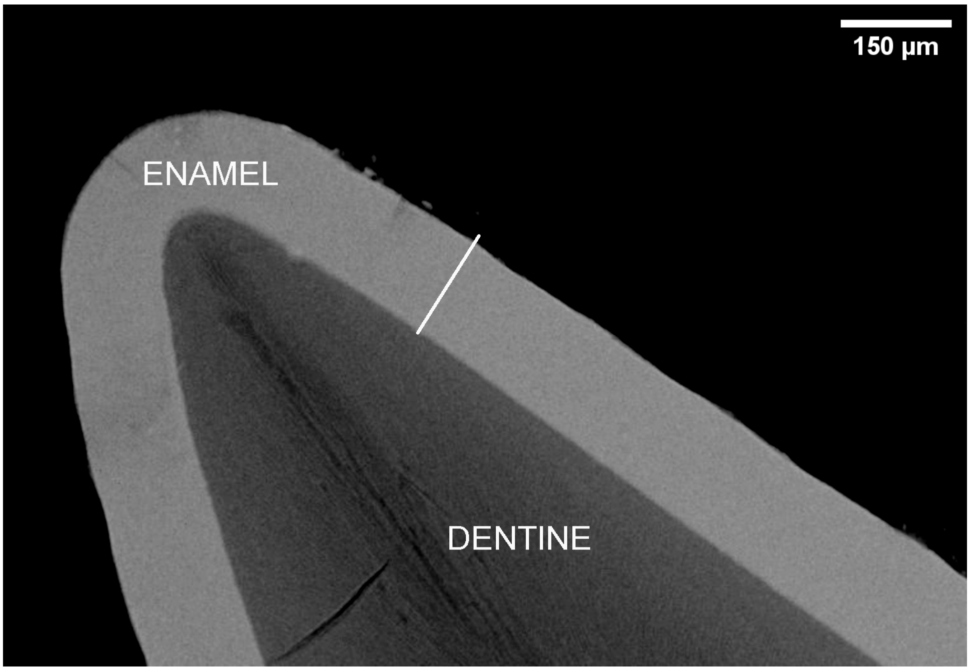

Figure S10. Microradiographic image of a ground section of dental enamel sample from *H. hydrochaeris*. Points of biochemical components (mineral, water, and organic) measurements (at 20, 45, 70, 95, and 120 µm from the enamel surface) were located along the longitudinal line shown in the enamel layer. The enamel thickness is 145 µm.

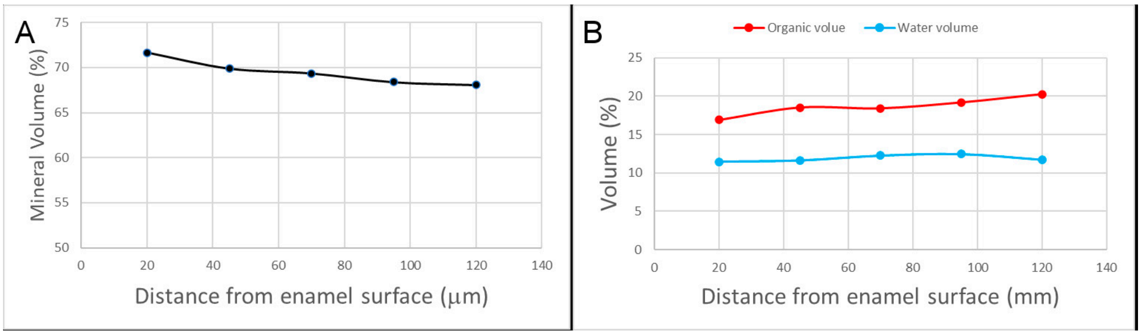

Figure S11. Mineral (A), organic (B), and total water (B) enamel component volumes of *H. hydrochaeris* at histological points located along the longitudinal line shown in Figure 10.
